# Levocetrizine attenuates cyclophosphamide-induced lung injury through inhibition of TNF-α, IL-1β, TGF-β and MMP-9

**DOI:** 10.1186/s40360-023-00717-3

**Published:** 2023-12-11

**Authors:** Dalia H. El-Kashef, Mona Abdel Rahim

**Affiliations:** 1 Department of Pharmacology and Toxicology, Faculty of Pharmacy, Mansoura University, Mansoura, 35516 Egypt; 2 Urology and Nephrology center, Faculty of Medicine, Mansoura University, Mansoura, Egypt

**Keywords:** Cyclophosphamide, Levocetrizine, TNF-α, IL-1β, TGF- β, MMP-9

## Abstract

Cyclophosphamide (CP) is an antineoplastic drug commonly used worldwide. Despite its spread, it causes fatal organ toxicity. Lung toxicity is a serious side effect of CP. Actually, in the past three years the world has been facing an un-predicted crisis following COVID-19 pandemic and the associated high-mortality rates attributed to respiratory distress. Accordingly; this study aimed to probe the potential prophylactic role of levocetrizine against CP-induced lung injury. Animals were allocated into three sets; control; CP and CP/Levo. CP was intraperitoneally injected in rats 150 mg/kg once on day 7. Levocetrizine was given orally for 14 days starting 7 days before CP injection. On the last day, all rats were sacrificed and lung tissues were kept for analysis. CP significantly elevated lung/body weight index, inflammatory cell counts, LDH, total protein, TNF-α, IL-1β, TGF-β and histamine levels in bronchoalveolar lavage (BAL). Moreover, it markedly increased expression of MMP-9 and contents of MDA, hydroxyproline, collagen and NOx besides decreasing GSH level and SOD activity in lung tissues. These biochemical results were further confirmed by histopathological examination. In contrast, treatment with levocetrizine significantly attenuated CP-induced pathological alterations. These findings propose that levocetrizine can attenuate CP-induced lung injury via exerting antioxidant, anti-inflammatory and anti-fibrotic effects.

## Introduction

Cyclophosphamide (CP) is a chemotherapeutic drug that plays an important role in immunosuppression. CP is used in treating many neoplastic diseases such as leukemia and lymphoma [1]. CP is metabolized via cytochrome P450 into phosphoramide mustard and acrolein. The therapeutic effect of CP is attributed to phosphoramide mustard but its side effects are ascribed to acrolein [2]. The later interferes with the oxidant/antioxidant balance and result in formation of reactive oxygen species (ROS). These ROS can cause lipid peroxidation of membranes and induce activation of several intracellular signaling pathways which in turn enhance formation of pro-inflammatory cytokines such as interleukin-1β (IL-1 β) and tumor necrosis factor-α (TNF-α) [3]. CP has been known to produce different forms of lung injury, from mild inflammation to fibrosis that is frequently life threatening [2]. Even though the functional and structural alterations associated with CP toxicity have been well elucidated, there is no effective therapy till now.

Histamine is a biogenic monoamine acting as a neurotransmitter and a signaling molecule. Also, histamine is a well-known inflammatory mediator so it is comprised in various physiological and pathological conditions. Histamine performs its action via interaction with four specific receptors (H_1_–H_4_ receptors) [4]. Histamine has been implicated in the progression of CP induced lung injury. This was confirmed by a previous study which reported that histamine was liberated from mast cells then trapped in lung tissue throughout the inflammation process and acted as reputed mediator in sequence of events that lead to fibrosis [3].

Levocetirizine is a selective, potent, oral histamine H_1_-receptor antagonist that is used in treatment of chronic urticaria and allergic rhinitis [5]. It has been reported to possess anti-inflammatory property in various allergy models through inhibition of multiple inflammatory mediators [6, 7].

This study aimed to scrutinize the possible prophylactic impacts of levocetrizine against CP-induced lung injury as well as the probable underlying mechanisms.

## Materials and methods

### Chemicals

Levocetrizine was obtained as a pharmaceutical product (Levcet tablets 5 mg, Marcyrl Co, Egypt). It was suspended in 0.5% carboxy methyl cellulose (CMC). CP was purchased as a commercial preparation (Endoxan vial 200 mg) from Baxter (Germany). It had been dissolved in 0.9% NaCl. All chemicals used were of the finest grade.

### Animals

Adult male SD rats (180 ± 20 g) were obtained from Vacsera, Helwan, Egypt. They were habituated for 7 days prior to the experimentation, placed in plastic cages, allowed free food and water; and temperature was accustomed at 23 ± 2 °C. Experimental procedures were carried out in accordance with the ethical guidelines adopted by “Research Ethics Committee, Faculty of Pharmacy, Mansoura University, Egypt” (code number: 2021 − 242).

### Experimental procedures

**Fig. 1:**
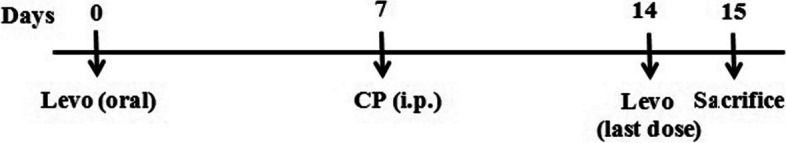
The animal protocol used to induce inflammation and fibrosis in lung of rats. *CP* Cuclophosphamide, *levo* Levocetrizine

Rats were arbitrarily allocated into 3 groups of 10 rats each; Control group; CP group, injected with CP (150 mg/kg/day, i.p.) once [8, 9] then left without treatment for 7 days; CP/Levo group, received levocetrizine (1 mg/kg, orally) [10] for 14 successive days and CP was single injected on the 7th day 1 h after receiving levocetrizine (Fig. 1).

On the last day, the weight of rats was recorded. The animals were euthanized and sacrificed by cervical dislocation after being anesthetized with secobarbital (50 mg/kg/*i.p*). Rats of each group were divided into 2 subgroups; bronchoalveolar lavage (BAL) fluid was collected from the first subgroup. In the other subgroup, the left lungs were used for assessment of oxidative stress biomarkers (malondialdehyde (MDA), reduced glutathione (GSH) superoxide dismutase (SOD)) and total nitric oxide (NOx) content. Right lungs were preserved in 10% formalin to undergo histopathological and immunohistochemical examination. The study was conducted in accordance with the Basic & Clinical Pharmacology & Toxicology policy for experimental and clinical studies [11].

### Calculation of lung/body weight index

In order to evaluate pulmonary edema, lung/body weight index was estimated. It was calculated by the following equation (lung weight ÷ rat body weight).

### Collection of BAL

BAL was collected via opening the thoracic cavity, cannulating the trachea, then the lung was washed with normal saline 3 times with 1 ml/wash. BAL was immediately centrifuged to get the cell pellet for the determination of total and differential cell count. Supernatants were used for assessment of lactate dehydrogenase (LDH), total protein content, TNF-α, IL-1β and TGF-β.

#### Estimation of LDH

LDH activity was assessed by Biomed assay kits (Cairo, Egypt) according to the instructions provided with the kit.

#### Assessment of total protein

Total protein content was estimated in BAL in order to evaluate the vascular permeability, via SPINREACT kit (Spain).

#### Estimation of inflammatory cytokines

TNF-α and IL-1β levels in BAL were evaluated using ELISA kits (RayBiotech, USA) following the manufacturer’s instructions.

#### Estimation of fibrotic marker

TGF-β level was measured using ELISA kit (Cusabio, USA) in accordance to the guidelines provided.

#### Determination of histamine

Histamine level was assayed using ELISA kit (Abcam ab213975). The assay was carried out in accordance to the provided instructions.

### Preparation of lung homogenates

Tissue homogenates (10% w/v) were obtained by homogenizing the left lungs in KCl (1.15%) to be used in assessment of oxidative stress and NOx content.

#### Measurement of MDA content

MDA estimation was used to reflex lipid peroxidation. In brief, MDA reacts with thiobarbituric acid to form a pink chromogen. The absorbance of that pink color was recorded at 532 nm [12].

#### Assessment of GSH level

GSH was estimated by a reaction of 5, 5’ dithiobis (2-nitrobenzoic acid DTNB) with GSH to obtain a yellow color measured spectrophotometrically at 412 nm [13].

#### Evaluation of SOD activity

It was determined by observing the SOD-inhabitable pyrogallol auto-oxidation [14].

#### Determination of NOx content

NOx content was assessed to indicate nitric oxide production as formerly demarcated [15].

### Estimation of hydroxyproline and collagen contents

Determination of hydroxyproline content reflects the degree of deposition of collagen within the lungs. The procedures were performed as defined previously [16]. Collagen content in the lung was calculated by the following equation (hydroxyproline content X 13.5) [17].

### Histopathology

Right lungs were entrenched in paraffin after preservation in formalin. These blocks were stained with H&E after being sliced (5 μm), then analyzed microscopically (magnification x100). Another set of slides was stained with Masson’s trichrome to evaluate fibrosis then examined under microscope. The severity of fibrosis was measured as previously defined [18]. The score of lung fibrosis was graded on a scale from 0 to 5 and the mean was estimated. In brief “Grade 0, no fibrosis; grade 1, mild thickening of the walls of alveoli or bronchioles; grade 5, severe fibrosis and damage to the lung architecture”.

### Immunohistochemistry

Formalin-fixed samples which were processed into paraffin blocks were sliced into 5 μm sections. The sections were hydrated and immersed in EDTA solution, PH8. Next, they were treated with 0.3% hydrogen peroxide and protein block. Afterward, they were incubated with primary antibody for matrix metalloproteinase-9 (MMP9) polyclonal antibody (PA5-13199, Invitrogen, 1:100 dilution). They were incubated with anti-mouse IgG secondary antibodies (EnVision + System HRP; Dako) for half an hour at 25 ^o^C after being rinsed with PBS. They were envisioned with commercial kits (Liquid DAB + Substrate Chromogen System; Dako), and lastly counterstained with Mayer’s hematoxylin and examined via light microscopy.

### Statistical analysis

Data were expressed as mean ± SEM. One-way ANOVA followed by Tukey’s multiple comparison test were performed for parametric data. Kruskal-Wallis test followed by Dunn’s multiple comparison test were applied for non-parametric data. Instat software (version 3) and Graphpad prism (version 5) were used. Significance was fixed at *p* < 0.05.

## Results

### Effect of levocetrizine on body weight and lung/body weight index of rats

Single injection of CP significantly reduced the animal body weight and increased lung body weight index upon comparison with normal rats. Levocetrizine treatment resulted in restoring body weights, decreasing pulmonary edema and reducing lung body weight index when compared to CP group (Table 1).

**Table 1:** Effect of levocetrizine on body weight and lung/body weight index of rats

Groups	Weight on day 7 (gm)	Final weight (gm)	Change in body weight	Lung/body weight index
**Control**	191 ± 3.1	220.4 ± 5.03	29.4 ± 3.1	5.5 ± 0.25
**CP**	190 ± 2.3	161.6 ± 1.50^*^	-28.2 ± 1.8^*^	8.2 ± 0.38^*^
**CP/Levo**	184 ± 6.6	177.3 ± 5.67^*$^	-7.8 ± 1.1^*$^	6.6 ± 0.24^$^

Values represent the mean ± SEM (*n* = 10, *n* = 5, respectively). * *p* < *0.05 vs.* control, $ *p* < *0.05 vs.* CP

### Effect of levocetrizine on inflammatory cell counts in BAL of rats

CP intoxication resulted in significant elevation in total and differential cell count in BAL relative to the control group. However, administration of levocetrizine significantly reduced these parameters upon comparison with CP group (Table 2).

**Table 2:** Effect of levocetrizine on inflammatory cell counts in BAL of rats

Groups	Total cell count × 10^4^	Neutrophil (cell/lung) × 10^4^	Lymphocytes (cell/lung) × 10^4^	Eosinophils (cell/lung) × 10^4^	Basophils (cell/lung) × 10^4^
**Control**	8.3 ± 0.6	5.1 ± 0.4	2.8 ± 0. 2	0. 1 ± 0.01	0. 11 ± 0.01
**CP**	93.7 ± 2.1^*^	67. 1 ± 2.3^*^	22. 4 ± 1^*^	2.3 ± 0. 4^*^	1.87 ± 0.04^*^
**CP/Levo**	41 ± 1.1^*$^	24.6 ± 0.5^*$^	14.9 ± 0.6^*$^	0.8 ± 0.02^$^	0. 54 ± 0.07^*$^

Values represent the mean of 5 rats ± SEM. * p < 0.05 vs. control; *$ p < 0.05 vs.* CP

### Effect of levocetrizine on LDH and total protein in BAL of rats

**Fig. 2:**
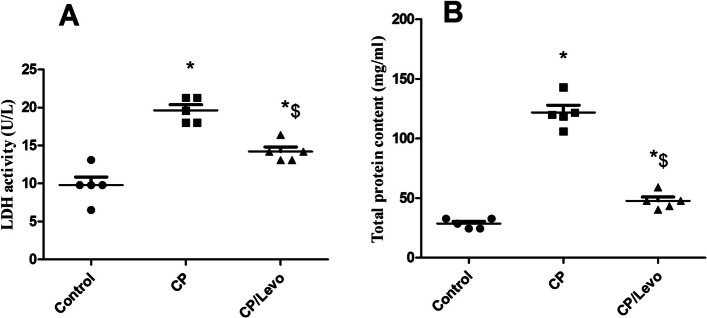
Effect of levocetrizine on LDH and total protein in BAL of rats. Values represent the mean of 5 rats ± SEM. * p < 0.05 vs. control; *$ p < 0.05 vs.* CP

Figure 2 showed that CP injection induced significant increase in LDH and total protein levels in BAL relative to the control group. Concomitant treatment with levocetrizine substantially decreased these elevated levels when compared to CP group.

### Effect of levocetrizine on TNFα and IL-1β in BAL of rats

Table 3 showed that single injection of CP significantly increased both TNF-α and IL-1β levels relative to the control group. Meanwhile, treatment with levocetrizine caused a profound decline in these levels relative to CP group.

**Table 3:** Effect of levocetrizine on TNFα and IL-1β in BAL of rats

Groups	TNF-α (pg/ml)	IL-1β (pg/ml)
**Control**	15.3 ± 1.2	18.4 ± 0.8
**CP**	38.6 ± 2.8^*^	49.1 ± 2.3^*^
**CP/Levo**	15.6 ± 1.5^$^	21.6 ± 0.6^$^

Values represent the mean of 5 rats ± SEM. * p < 0.05 vs. control; *$ p < 0.05 vs.* CP

### Effect of levocetrizine on TGF-β in BAL of rats

**Fig. 3:**
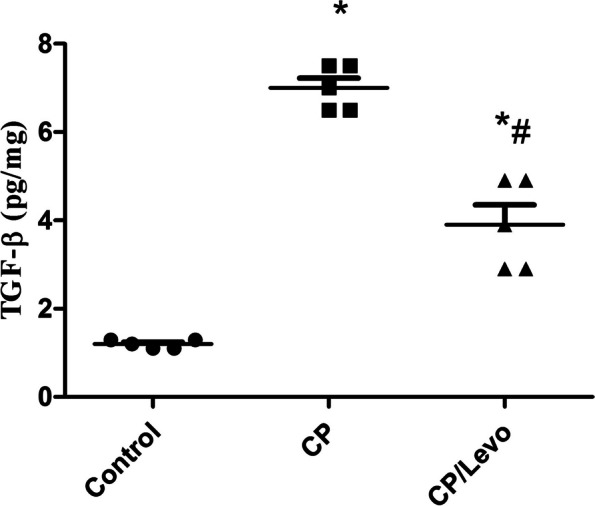
Effect of levocetrizine on TGF-β in BAL of rats. Values represent the mean of 5 rats ± SEM. * p < 0.05 vs. control; *$ p < 0.05 vs.* CP

Single injection of CP produced marked increase in levels of TGF-β in comparison with control group. CP/Levo group revealed a marked reduction in these levels compared to the diseased group (Fig. 3).

### Effect of levocetrizine on histamine in BAL of rats

**Fig. 4:**
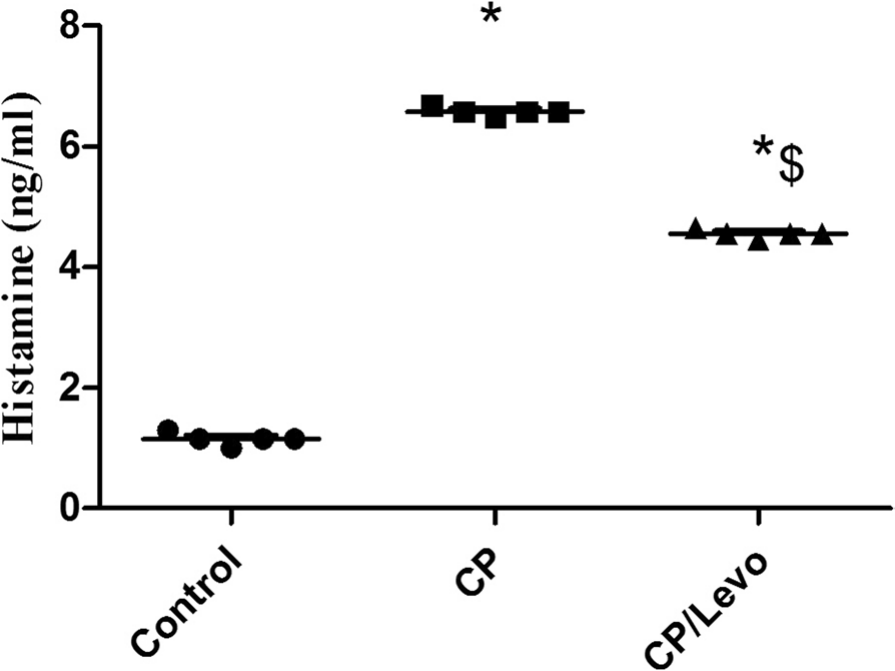
Effect of levocetrizine on histamine in BAL of rats. Values represent the mean of 5 rats ± SEM. * p < 0.05 vs. control; *$ p < 0.05 vs.* CP

Figure 4 clarified that CP markedly increased levels of histamine upon comparison with control group. Treatment with levocetrizine significantly decreased these levels upon comparison with CP group.

### Effect of levocetrizine on antioxidant status

**Fig. 5:**
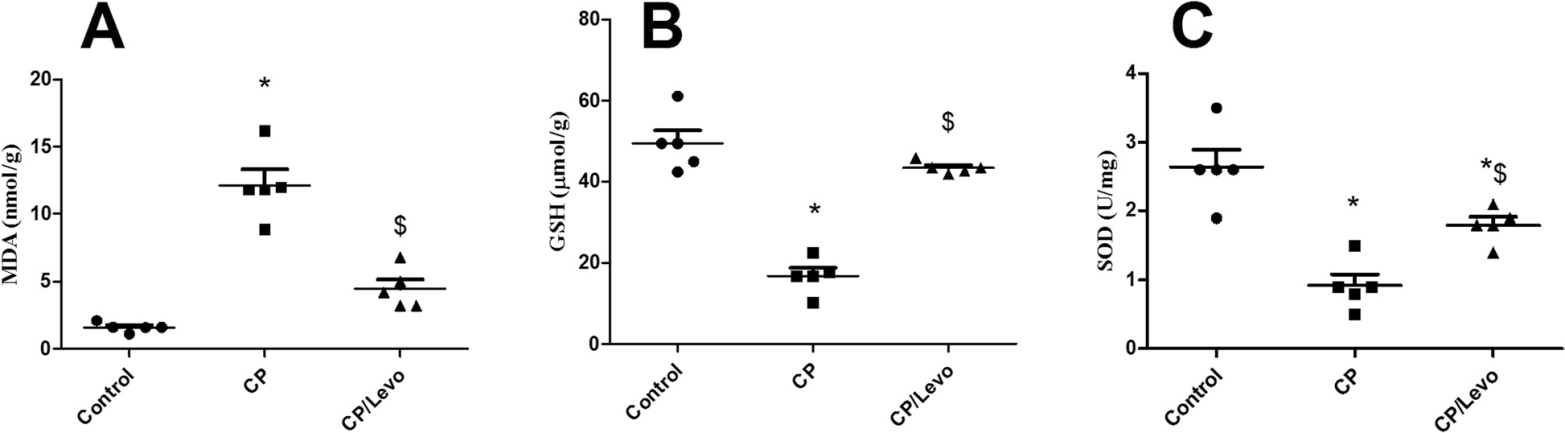
Effect of levocetrizine on antioxidant status. Values represent the mean of 5 rats ± SEM. * p < 0.05 vs. control; *$ p < 0.05 vs.* CP

Figure 5 showed that CP injection significantly increased pulmonary levels of MDA and reduced pulmonary GSH and SOD levels when compared to normal rats. By contrast, these observations were reversed upon administration of levocetrizine.

### Effect of levocetrizine on NOx content

**Fig. 6:**
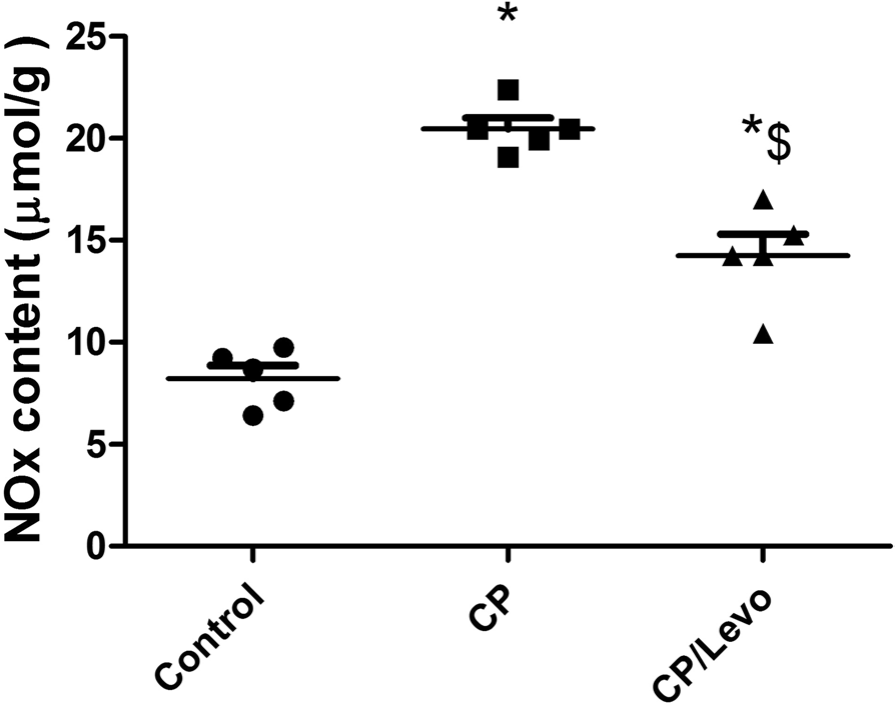
Effect of levocetrizine on NOx content. Values represent the mean of 5 rats ± SEM. * p < 0.05 vs. control; *$ p < 0.05 vs.* CP

Single injection of CP induced significant elevation in NOx content when compared to the control group. Oral administration of levocetrizine inhibited that increase upon comparison with CP group (Fig. 6).

### Effect of levocetrizine on hydroxyproline and collagen content

CP markedly increased contents of hydroxyproline and collagen upon comparison with normal rats. Pretreatment with levocetrizine markedly reversed that increase upon comparison with CP group (Table 4).

**Table 4:** Effect of levocetrizine on hydroxyproline and collagen contents

Groups	Hydroxyproline (mg/g tissue)	Collagen (mg/g tissue)
**Control**	27.1 ± 0.15	353.2 ± 1.9
**CP**	50.5 ± 0.94^*^	656.5 ± 12.3^*^
**CP/Levo**	40.5 ± 0.80^*$^	527.4 ± 10.3^*$^

Values represent the mean of 5 rats ± SEM. * p < 0.05 vs. control; *$ p < 0.05 vs.* CP

### Effect of levocetrizine on histopathology

**Fig. 7:**
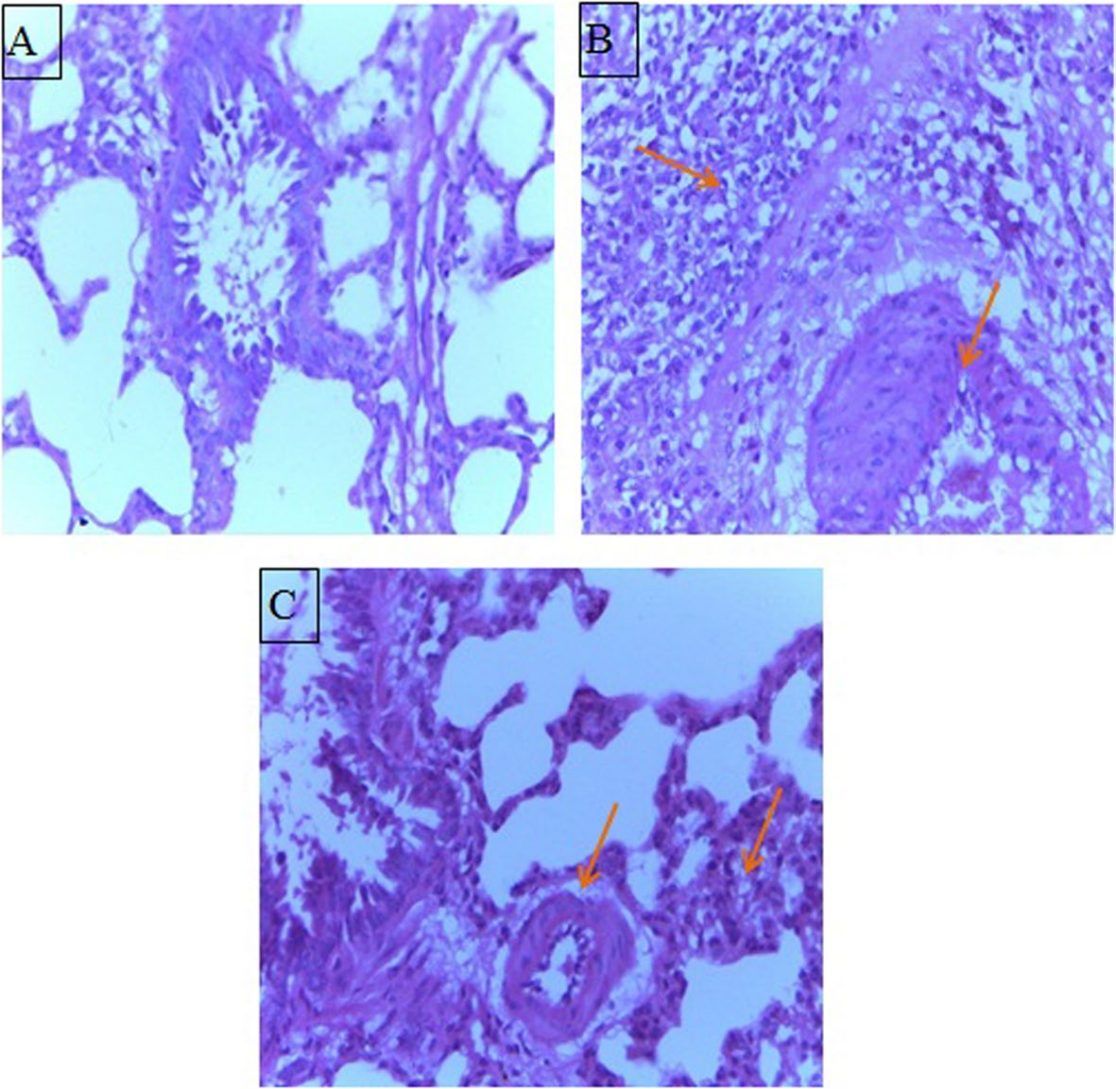
Effect of levocetrizine on histopathology (HE X 400). **A** Control group: showed no signs of inflammation. **B** CP group: showed marked inflammation showed by arrow, the blood vessels were dilated and showed inflammation. **C** CP/Levo showed mild inflammatory response and the vessels were unremarkable

**Fig. 8:**
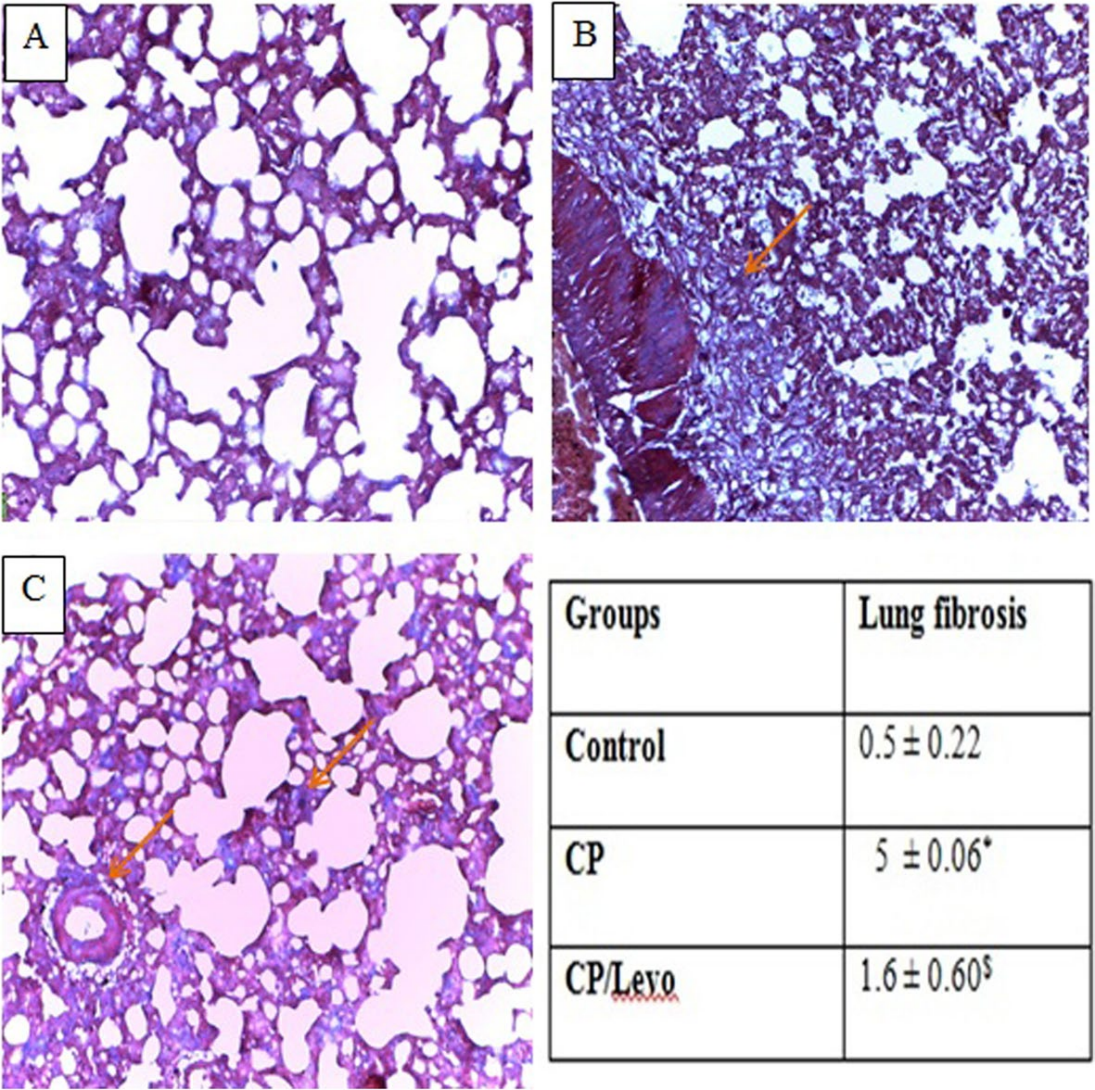
Effect of levocetrizine on histopathology (Masson’s trichrome ×200). **A** The control group showed normal alveolar wall with no evidence of fibrosis. **B** CP group showed focal alveolar damage and broncheoalveolar walls were thickened by fibrosis (arrow). **C** Cp / Levo showed minimal fibrotic changes (arrows). **D** Lung fibrosis score. Values represent the mean of 5 experiments ± SEM. * *p* < *0.05 vs.* control, $ *p* < *0.05 vs.*. CP (Kruskal Wallis)

Sections of control group bared normal lung structure. Rats that are injected with CP revealed lung damage indicated by increased inflammatory response, colossal number of macrophages, thickened alveolar walls and fibrosis. Treatment with levocetrizine significantly alleviated the CP-induced damage to the lung tissue (Figs. 7 and 8).

### Effect of levocetrizine on immunohistochemistry

**Fig. 9:**
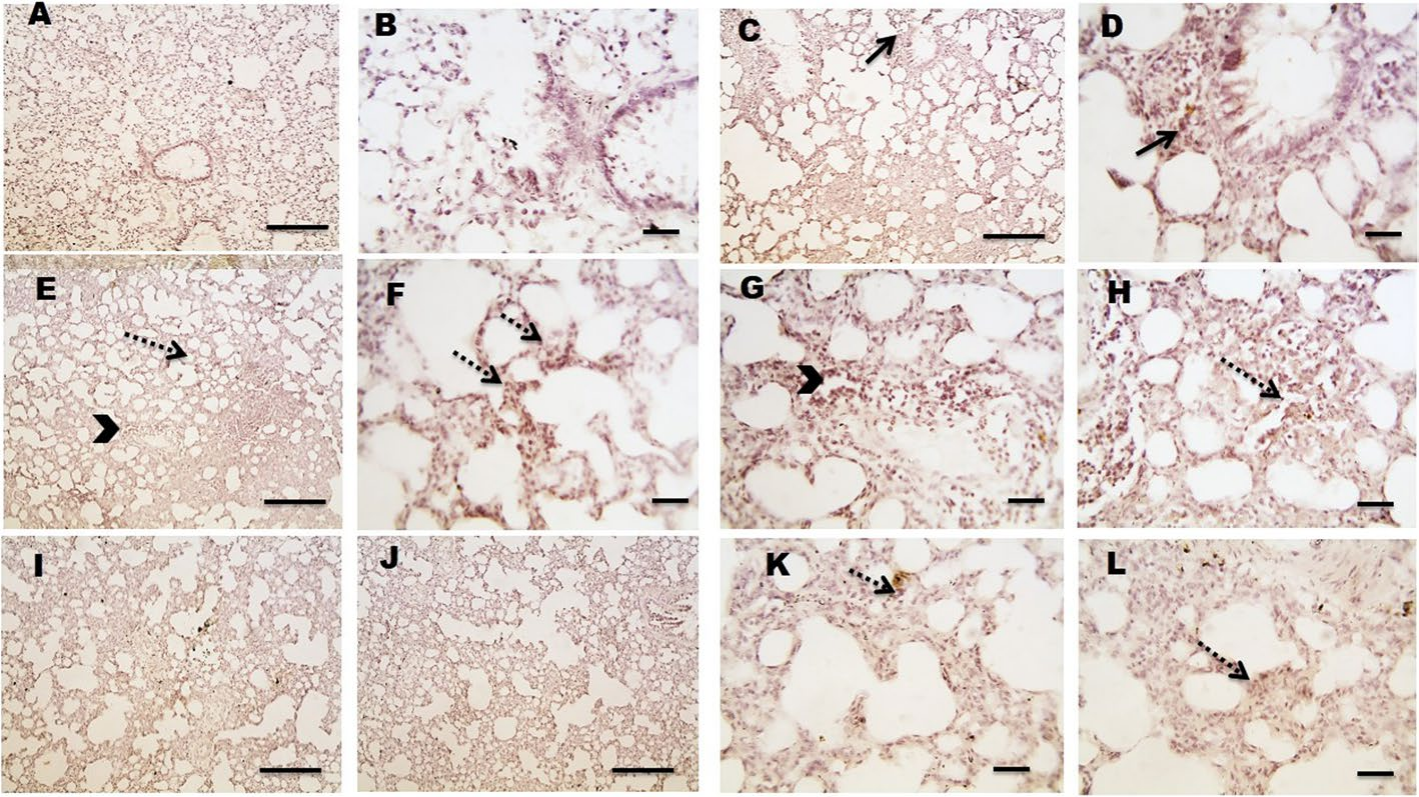
Effect of levocetrizine on MMP-9 (IHC). **A** and **B**, Control group: showed negative immunostaining. **C**–**H**, Cyclophosphamide (CP) group showed: positive brown expression against MMP-9 in leukocytes infiltrating in peribronchial (arrows), perivascular (arrowheads) and in interstitial tissues (dashed arrows) I-L, Levocetrizine group: very mild positive brown expression against MMP-9 that appears in interstitial tissues (dashed arrows). IHC counterstained with Mayer’s hematoxylin. Low magnification X:100 bar 100 (A, C, E, I, J) and high magnification X:400 bar 50 (B, D, F, G, H, K, L)

Micrographs of lung sections against MMP-9 showed absent expression in the control group (Fig. 9A&B). Lung sections from diseased group showing prominent positive brown expression against MMP-9 in leukocytes infiltrating in peribronchial (arrows), perivascular (arrowheads) and in interstitial tissues (dashed arrows) (Fig. 9C H). Lung sections from levocetrizine-treated group showing very mild positive brown expression against MMP-9 that appears in interstitial tissues (dashed arrows) (Fig. 9I L).

## Discussion

CP is widely used in treatment of many types of cancer; however it exerts serious side effects including lung toxicity. CP-induced functional and structural changes in experimental animals resemble those observed in humans [3]. This study purposed to explore the protective impact of antihistamine, levocetrizine, against lung toxicity induced by CP.

Our data showed that single injection of CP significantly decreased the animal body weight, and caused a profound elevation in the lung/body weight index in comparison with the control rats. That decrease in body weight might be owed to the cytotoxic effect of CP, and the rise in lung/body weight indices might be related to pulmonary edema. Treatment with levocetrizine significantly reversed the loss in animal body weight and decreased the elevated lung/body weight indices.

In this study, CP intoxicated rats exhibited marked elevation in inflammatory cells counts in BAL in comparison with the control group. This finding agreed with previous studies [8, 19]. Administration of levocetrizine markedly decreased the infiltration of inflammatory cells into the lung tissue evidenced by reduced total and differential cell counts in BAL. This effect might be due to ability of levocetrizine to counteract the inflammatory reactions [5].

Injection of CP produced significant increase in levels of LDH and total protein in BAL. This increase might be ascribed to their release into the blood stream after damage of the plasma membrane caused by the production of ROS following CP exposure [9]. Levocetrizine administration reduced the elevated LDH and total protein levels in BAL indicating notable decrease in lung permeability.

ROS overproduction, lipid peroxidation and decline in the antioxidant defense mechanism are involved in pathogenesis of CP-induced lung toxicity. In accordance with earlier researches [3, 19], our results showed that CP injection induced oxidative stress. This was proved by the substantial increase in MDA along with reduction in GSH and SOD levels in lung tissue. That decrease in SOD activity might be due to overproduction of ROS while depletion of GSH was formerly clarified by conjugation of CP and acrolein (its metabolite) directly with -SH groups either in free or bound state [19, 20]. Levocetrizine treatment showed a notable anti-oxidant effect as it markedly reduced MDA levels and restored GSH and SOD levels in lung tissues. The ability of levocetrizine to elevate GSH is an important mechanism in protection against CP-induced injury. One of the probable mechanisms which might arbitrate the antioxidative properties of levocetirizine is its ability to block H_1_ receptor. Histamine has been reported to stimulate hydrogen peroxide release by bronchial epithelial cells through H_1_ receptor-dependent signaling [21]. Several H_1_-antihistamines diminished the generation of ROS in neutrophils isolated from rat blood [22]. Furthermore, levocetirizine might inactivates ROS through activation of SOD, the only antioxidant enzyme that can scavenge superoxide or via direct scavenging activity [23]. In addition, levocetirizine might mitigate generation of oxidants via dropping alveolar infiltration of inflammatory cells. Correspondingly, levocetirizine inhibited recruitment of inflammatory cells and suppressed inflammation in a model of lipopolysaccharide-induced lung inflammation [10].

Nitric oxide (NO) has been stated to play a crucial role in CP-induced toxicity [24]. In this study, CP injection induced significant increase in NOx levels confirming our previous study which declared that elevation of NOx might be attributed to up regulation of iNOS [8]. NO might react with superoxide anions and form peroxynitrite, and up regulate proinflammatory cytokines production.

Several cytokines such as TNF-α and IL-1β have a pivotal role in airway inflammation [2]. TNF- α is responsible for inflammation and fibrosis that occur in the lung after exposure to toxicants, which prompts death signaling and provokes alveolar dysfunction [25]. IL-1β is produced by activated macrophages and is implicated in many cellular events, comprising cell proliferation and differentiation [26]. The present data showed that CP intoxication caused marked elevation in levels of inflammatory cytokines, TNF-α and IL-1β. This observation agreed with our preceding work that proposed that inflammation might owe to recruitment of neutrophils and macrophages which cause the release of free radicals and proteolytic enzymes and finally lead to connective tissue damage [8]. Levocetrizine successfully lowered the levels of these cytokines in comparison with the diseased group. This observation confirmed the results of previous studies that admitted the anti-inflammatory effect of levocetrizine [5, 27].

This study showed that rats injected with CP revealed lung fibrosis, as evidenced by marked increase in the TGF-β levels. TGF-β has been stated to play a crucial role in the progression of fibrosis via stimulating the activated fibroblasts to produce collagen and fibronectin, and through inhibiting proteases which lyse the extracellular matrix (ECM) [3, 28]. It has been previously reported that continuous formation of myofibroblasts and abnormal MMPs can lead to deposition of ECM. Accordingly, ECM substitutes the normal structure disturbing the organ normal function. Hence, major snags are related to the imbalance between ECM formation and degradation [29]. Our results revealed marked expression of MMP-9 in CP group. Levocetrizine treatment significantly reduced the elevated levels of TGF-β and resulted in profound decrease in expression of MMP-9 in lung tissues. In line with our study, levocetrizine has been reported to avert the up regulation of fibrogenic markers in patients with chronic rhinosinusitis with nasal polyps [30].

Histopathological evaluation of lungs supported the biochemical findings where sections of CP group exhibited marked inflammation and fibrosis. This result came line with earlier reports [8, 19]. By contrast, levocetrizine effectively aborted these histopathological alterations in lung tissue suggesting its potential to mitigate CP-induced lung injury.

**Fig. 10:**
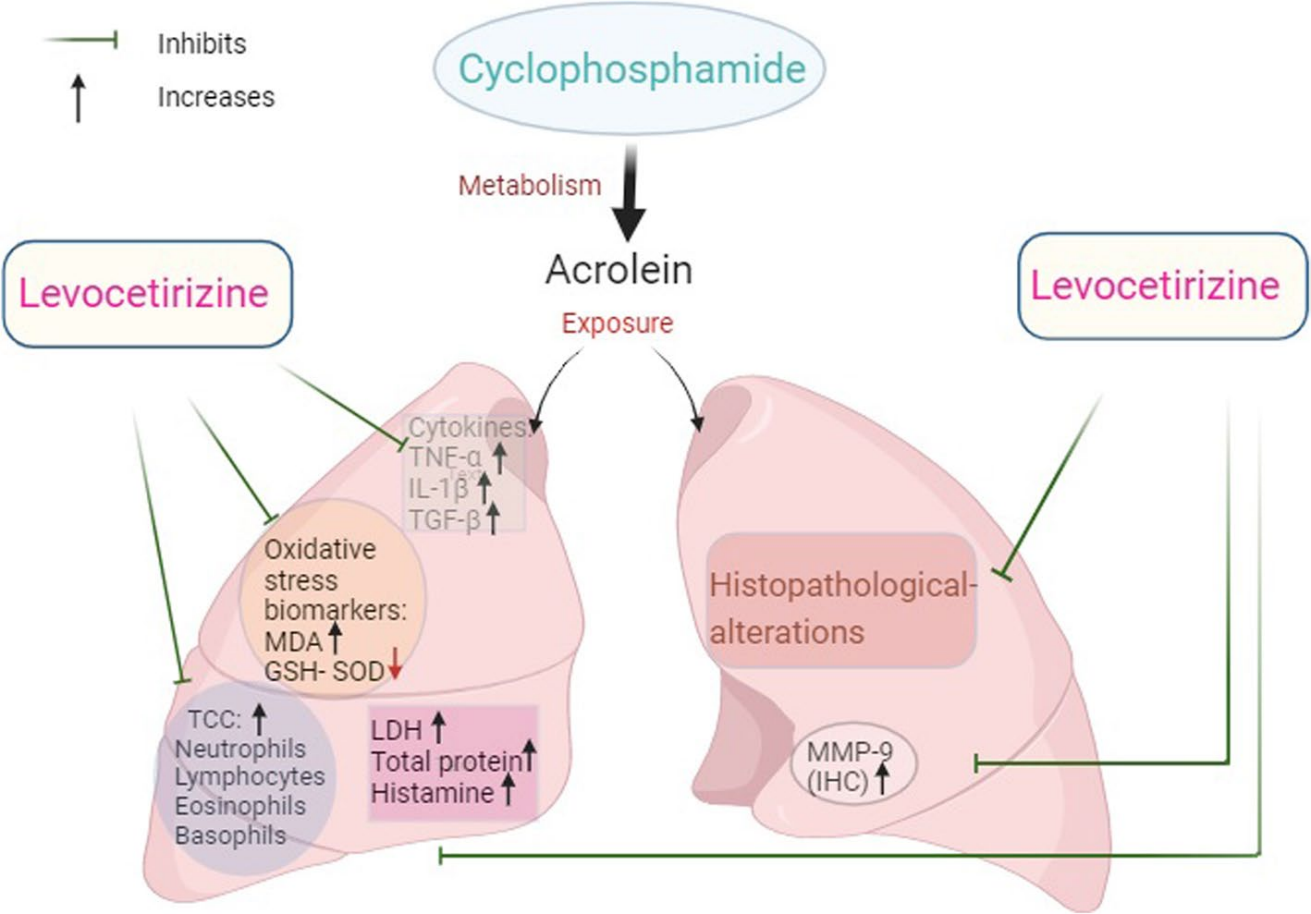
Schematic presentation of cyclophosphamide-induced lung injury and amelioration by levocetrizine

In conclusion, these results propose that levocetrizine exerts protective effects against CP-induced pulmonary toxicity. These effects might be attributed to its capability to alleviate oxidative stress, inflammatory and fibrotic responses (Fig. 10). Thus, levocetrizine may have clinical importance in preventing CP-induced lung injury after further confirmatory studies.

## Data Availability

The datasets used and/or analyzed during the current study are available from the corresponding author on reasonable request.
